# Association of Multiple Sclerosis Susceptibility Variants and Early Attack Location in the CNS

**DOI:** 10.1371/journal.pone.0075565

**Published:** 2013-10-10

**Authors:** Ellen M. Mowry, Robert F. Carey, Maria R. Blasco, Jean Pelletier, Pierre Duquette, Pablo Villoslada, Irina Malikova, Elaine Roger, R. Phillip Kinkel, Jamie McDonald, Peter Bacchetti, Emmanuelle Waubant

**Affiliations:** 1 Multiple Sclerosis Center, Department of Neurology, Johns Hopkins University, Baltimore, Maryland, United States of America; 2 Multiple Sclerosis Center, Department of Neurology, University of California San Francisco, San Francisco, California, United States of America; 3 Department of Neurology, Hospital Universitario Puerta de Hierro, Madrid, Spain; 4 Pole de Neurosciences Cliniques, Service de Neurologie, Centre de Résonance Magnétique Biologique et Médicale, Centre Hospitalier Universitaire Timone, Aix Marseille Université, Marseille, France; 5 Multiple Sclerosis Clinic, Centre Hospitalier de L'Universite de Montreal, Montreal, Canada; 6 Center of Neuroimmunology, Institute of Biomedical Research August Pi Sunyer-Hospital Clinic, Barcelona, Spain; 7 Department of Neurology, Beth Israel Deaconess Medical Center, Harvard Medical School, Boston, Massachusetts, United States of America; 8 Department of Epidemiology and Biostatistics, University of California San Francisco, San Francisco, California, United States of America; University of Oxford, United Kingdom

## Abstract

**Objective:**

The anatomic location of subsequent relapses in early multiple sclerosis (MS) appears to be predicted by the first attack location. We sought to determine if genetic polymorphisms associated with MS susceptibility are associated with attack location.

**Methods:**

17 genome-wide association study-identified MS susceptibility polymorphisms were genotyped in 503 white, non-Hispanic patients seen within a year of MS onset. Their association with the CNS location of the first two MS attacks was assessed in multivariate repeated measures analyses (generalized estimating equations with robust standard errors).

**Results:**

The *IL12A* polymorphism was independently associated with increased odds of attacks involving the spinal cord (OR = 1.52, 95% CI 1.11, 2.07, p = 0.009), as was the IRF8 polymorphism (OR = 2.40, 95% CI [1.04, 5.50], p = 0.040). The *IL7R* polymorphism was associated with reduced odds of attacks involving the brainstem/cerebellum (OR = 0.46, 95% CI 0.22, 0.97, p = 0.041), as were the *TNFRSF1A* and *IL12A* polymorphisms. The *CD6* polymorphism conferred reduced odds of optic neuritis as an attack location (OR = 0.69, 95% CI [0.49, 0.97], p = 0.034). Several other genes showed trends for association with attack location.

**Conclusions:**

Some of the MS susceptibility genes may be associated with MS attack location. The *IL12A* polymorphism is of particular interest given that interferon beta therapy appears to influence IL12 levels. These findings may lead to improved understanding of MS pathogenesis and treatment.

## Introduction

While the anatomic location of multiple sclerosis (MS) attacks differs from one person to the next, we have recently demonstrated that an individual patient's early attacks are likely to occur in the same location within the central nervous system (CNS) [1]. In other words, a person who has spinal cord involvement in the first attack is likely to have involvement thereof during the second attack, whereas a person with onset involving the optic nerve has greater odds of experiencing optic neuritis during the second attack. We hypothesized that genes known to be associated with MS susceptibility may also determine various features of its phenotype. In this investigation, we sought to determine if MS susceptibility genes are associated with the CNS location of early MS attacks.

## Methods

### Ethics statement

This study was approved by the University of California, San Francisco Committee on Human Research (CHR); each center contributed data from patients enrolled in Institutional Review Board-approved studies who had provided written informed consent.

### Subjects and sites

This is a retrospective study of white patients with clinically isolated syndrome or relapsing-remitting MS. Race was restricted to reduce genetic heterogeneity. Subjects at five MS centers (UCSF, San Francisco, USA; MS Unit, Marseille, France; Centre Hospitalier de L'Université de Montreal, Montreal, Canada; University of Navarra, Spain; Hospital Puerta de Hierro Neuroimmunology Unit, Madrid, Spain); and from two clinical trials (Controlled High Risk Avonex Multiple Sclerosis Prevention Study in Ongoing Neurologic Surveillance [CHAMPIONS] and Optic Neuritis Treatment Trial [ONTT])[2], [3] who were seen within a year of their first attack and who had brain magnetic resonance imaging (MRI) within six months of symptom onset were included. At UCSF, three different cohorts were evaluated. The first was the UCSF MS Center database, in which data for all patients (children and adults) seen in the first year of MS onset are entered. Routine clinic visits for UCSF patients typically take place every 6 months. Subjects from two trials (the atorvastatin and riluzole trials) were also included [4], [5]. Atorvastatin participants were seen within 90 days of onset and were evaluated monthly for three months and then quarterly (total duration of follow-up was 18 months). For the riluzole study (which was incomplete when the data were frozen for this analysis), patients were enrolled in the first year of MS onset; they were evaluated monthly for six months and then quarterly for two to three years. In all three cohorts, additional visits were scheduled for suspected relapses. At Aix Marseille Université, records of patients seen within six months of disease onset who participated in a prospective longitudinal study of MS that began in 2000 were retrieved from the EDMUS database [6]. In this cohort, patients had clinic visits and MRIs every 3 months for a year, then every 6 months for 3 years, and annually thereafter. Patients at L'Université de Montreal, Canada were offered participation in an observational study from September, 2007 until March, 2009. Visits occurred at least once a year but were more frequent if the disease was active. Patients evaluated at the University of Navarra, Spain were enrolled at the time of MS onset in a prospective MS biomarker study (using the EDMUS database) that began in 2001 and were clinically evaluated every 3 to 6 months thereafter [7]. At the Universitario Puerta de Hierro, CIS patients evaluated within a year of symptom onset had a neurologic examination at three months and were followed biannually thereafter, with additional visits for suspected relapses. The Optic Neuritis Treatment Trial (ONTT) enrolled participants within 8 days of optic neuritis onset [8]. Because some had had prior symptoms, only those without symptoms consistent with a previous MS/CIS attack were included. The participants were re-evaluated on days 4, 15, and 30, weeks 7, 13, and 19, months 6 and 12, and then annually [3]. The Controlled High Risk Avonex Multiple Sclerosis Prevention Study in Ongoing Neurologic Surveillance (CHAMPIONS) study was an extension study (open-label) of Controlled High Risk Subjects Avonex Multiple Sclerosis Prevention Study (CHAMPS) [2], [9]. CHAMPS enrolled subjects with a first attack if they were evaluated within 27 days of the onset of the neurologic symptoms and had an abnormal brain MRI (at least 2 lesions consistent with MS that had no corresponding clinical symptoms). Participants were randomized to intramuscular interferon beta-1a (weekly) versus placebo. CHAMPIONS enrolled CHAMPS subjects who gave consent into an open-label follow-up study. Subjects were evaluated biannually in both studies and within a week (CHAMPS) or two weeks (CHAMPIONS) of the onset of new symptoms concerning for relapse.

### Outcomes

Relapses, by definition, were new or recurring neurological symptoms that were referable to the CNS and lasted for at least 48 hours after a period of at least 30 days since the prior attack in the absence of fever or known infection. Transient recurrence of neurologic symptoms that began in the context of infection or fever was considered a pseudoexacerbation and was excluded. Based on clinical history and examination, each patient's relapses were coded as occurring in the spinal cord, brainstem/cerebellum, optic nerve, or cerebrum.

### Predictors

The predictors of interest were 16 non-HLA genetic polymorphisms that had been validated for MS susceptibility as of 2010 (Table S1 in File S1) [10]–[20]. In the UCSF laboratory of Dr. Jorge Oksenberg, TaqMan SNP genotyping assays were used for genotyping (Applied Biosystems Inc., Foster City, CA, USA). Each PCR reaction contained 10 ng of DNA, 1 x TaqMan Genotyping Master Mix, and 1 x SNP assay (both from Applied Biosystems, Inc.). Amplification was performed with an ABI 97000 GeneAmp PCR system (Applied Biosystems, Inc.). The PCR program used was: 95° Celsius for 10 minutes followed by 50 cycles of 95° Celsius for 15 seconds and then 62° Celsius for one minute. The plates were read with an ABI prism 7900HT Sequence Detection System (with SDS 2. software; Applied Biosystems, Inc.). *DRB1* genotyping was performed using a PCR locus-specific amplification, as previously described [19].

### Statistics

Summaries are given as percentages for categorical variables and as mean ± standard deviation (SD) or median (interquartile range) for continuous variables. For each of the four attack locations, a dichotomous outcome variable was defined as any involvement of the region versus no involvement. The presence of at least one copy of each risk allele versus no copies of the allele was assessed for its association with attacks involving each CNS location using generalized estimating equations with robust standard errors. This repeated measures approach was used to account for within-individual correlations and to take into account the location data from both the first and second attack in the same model. All of the non-HLA susceptibility genes, as well as *HLA-DRB1* status, were included in the models so that the independent effect of each gene could be determined. We also looked for interactions (with strong evidence defined by *p* value for interaction term <0.1) between *HLA-DRB1* and *EVI5* and *HRA-DRB1* and *CD226*, because such interactions have been identified in the MS susceptibility studies [15], [17].

## Results

### Patient and attack characteristics

We identified 503 patients (359 [71%] were women) seen within one year of MS onset (n = 199 from UCSF; n = 43 from Marseille; n = 36 from Montreal; n = 13 from Pamplona; n = 60 from Madrid; n = 68 from CHAMPIONS; n = 84 from ONTT). The average age of onset was 33±9 years; median disease duration was 46 days (interquartile range: 1, 351). Nearly all (459; 91%) patients had an abnormal brain MRI at baseline. On average, there were 7.5 (±5.2) years of follow-up. During the first attack, the following number of subjects had involvement of each location: spinal cord (207 [41%]); brainstem/cerebellum (126 [25%]); optic nerve (198 [39%]); cerebrum (12 [2%]). First attack location could not be definitively ascertained for 3 individuals. Of the 349 patients who had a second attack, a definite location could be ascribed for 302 attacks; of these, 171 (58%) had spinal cord involvement, 74 (25%) had brainstem/cerebellar involvement, 61 (21%) had optic nerve involvement, and 7 (2%) had cerebral involvement. The location of 45 of the 58 second attacks experienced by ONTT subjects could not be ascertained; second attack location could not be definitively ascertained for 2 additional individuals. Because some patients experienced attacks in more than one CNS location (and because attack location was missing for other participants), the percentages do not add up to 100% (and the numbers don’t add up to the total number of attacks). Because so few subjects had cerebral attacks, we were unable to assess it as an outcome. Disease-modifying therapy (DMT) was started in 345 patients (69%) at some point in the follow-up period, but only 134 (27%) had received treatment for the 90 days immediately preceding the second attack or the end of follow-up (if no second attack occurred).

Details of genotype frequencies are provided in Table S1 in File S1. One gene (*IRF8*) in one patient failed genotyping. There were no subjects without at least one copy of the *TYK2* G allele, so for this gene we assessed if two G alleles versus one was associated with attack location. No subject had more than one copy of the *TNFRSF1A* T allele; as such, we evaluated if having one versus no copies of the T allele was associated with attack location. Approximately half (n = 244; 49%) of the subjects had at least one copy of HLA-DRB1*1501 (the risk allele).

### Predictors of spinal cord involvement

The multivariate associations of the susceptibility polymorphisms with spinal cord involvement during the first two attacks are presented in Table? 1. The *IL12A* polymorphism was associated with greater odds of spinal cord attacks (OR 1.52, 95% CI [1.11, 2.07], p = 0.009). *IRF8* was also associated with greater odds of spinal cord involvement (OR = 2.40, 95% CI [1.04, 5.50], p = 0.040). There were trends for associations of *TNFRSF1A* and *TMEM39A* with increased odds of spinal cord involvement and of *IL2RA* and *GPC5* with reduced odds thereof. There was no strong evidence for interaction of *HLA-DRB1* with *EVI5* (p value for interaction term = 0.28) or with *CD226a* (p value for interaction term = 0.66). The estimates of association were similar in the univariate models (Table S2 in File S1).

**Table 1:** Predictors of attack location (multivariate models).

	Spinal cord	Brainstem/cerebellum	Optic nerve
*IL12A*	**1.52 (1.11, 2.07), p = 0.009**	0.72 (0.51, 1.02), p = 0.068	0.85 (0.60, 1.20), p = 0.36
*TMEM39A*	2.10 (0.83, 5.33), p = 0.12	3.11 (0.39, 24.81), p = 0.29	0.21 (0.04, 1.17), p = 0.075
*IL7R*	1.29 (0.61, 2.70), p = 0.51	**0.46 (0.22, 0.97), p = 0.041**	1.09 (0.53, 2.24), p = 0.82
*IL2RA*	0.59 (0.29, 1.20), p = 0.15	1.30 (0.58, 2.89), p = 0.52	0.88 (0.38, 2.02), p = 0.76
*CD6*	1.16 (0.85, 1.58), p = 0.36	1.15 (0.82, 1.61), p = 0.41	**0.69 (0.49, 0.97), p = 0.034**
*MPHOSPH9*	0.95 (0.69, 1.30), p = 0.75	1.26 (0.90, 1.76), p = 0.18	0.77 (0.55, 1.09), p = 0.14
*TNFRSF1A*	1.50 (0.91, 2.47), p = 0.11	0.55 (0.30, 1.02), p = 0.056	0.91 (0.51, 1.62), p = 0.75
*IRF8*	**2.40 (1.04, 5.50), p = 0.040**	0.70 (0.33, 1.51), p = 0.36	0.76 (0.31, 1.86), p = 0.55
*GPC5*	0.73 (0.53, 1.00), p = 0.051	1.02 (0.71, 1.46), p = 0.92	1.24 (0.88, 1.76), p = 0.22
*HLA-DRB1*	1.02 (0.75, 1.39), p = 0.88	1.03 (0.73, 1.45), p = 0.85	1.06 (0.75, 1.49), p = 0.75
*CD58*	0.54 (0.16, 1.79), p = 0.32	0.79 (0.25, 2.49), p = 0.69	1.33 (0.38, 4.64), p = 0.66
*RGS1*	1.44 (0.53, 3.94), p = 0.47	0.51 (0.16, 1.66), p = 0.27	0.84 (0.28, 2.51), p = 0.75
*EVI5*	0.96 (0.69, 1.34), p = 0.80	1.19 (0.82, 1.73), p = 0.36	0.80 (0.56, 1.15), p = 0.23
*KIF21B*	1.46 (0.76, 2.78), p = 0.25	1.03 (0.51, 2.10), p = 0.93	0.86 (0.44, 1.68), p = 0.65
*CLEC16A*	0.99 (0.58, 1.71), p = 0.98	0.79 (0.47, 1.32), p = 0.36	1.19 (0.68, 2.10), p = 0.54
*CD226a*	1.04 (0.73, 1.47), p = 0.83	0.98 (0.67, 1.41), p = 0.89	1.11 (0.76, 1.62), p = 0.58
*TYK2*	1.45 (0.61, 3.42), p = 0.40	0.96 (0.38, 2.39), p = 0.92	0.67 (0.30, 1.47), p = 0.32

Results presented as odds ratios (95% confidence intervals), p values. The analyses take into account first and second attack locations. Of the entire cohort (n = 503), 349 had a second attack. Attack location could not be resolved for 3 initial attacks and 47 second attacks.

### Predictors of brainstem/cerebellum involvement

In the multivariate model, the *IL7R* polymorphism was associated with lower odds of brainstem/cerebellar involvement (OR 0.46, 95% CI [0.22, 0.97], p = 0.041). The *IL12A* (OR 0.72, 95% CI [0.51, 1.02], p = 0.068) and *TNFRSF1A* (OR 0.55, 95% CI [0.30, 1.02], p = 0.056) polymorphisms tended to be associated with reduced odds of involvement of the brainstem/cerebellum, while the opposite was true of the *MPHOSPH9* polymorphism, although the confidence intervals included 1.0. Again, there was no strong evidence for interaction of *HLA-DRB1* with *EVI5* (p value for interaction term = 0.89) or with *CD226a* (p value for interaction term = 0.17). The univariate models had similar results (Table S2 in File S1).

### Predictors of optic nerve involvement

The *CD6* polymorphism was associated with reduced odds of optic neuritis during the first two attacks (OR = 0.69, 95% CI [0.49, 0.97], p = 0.034). There were trends for associations of the *TMEM39A* and *MPHOSPH9* polymorphisms with reduced odds of optic neuritis as well. There was no strong evidence for interaction of *HLA-DRB1* with *EVI5* (p value for interaction term = 0.48) or with *CD226a* (p value for interaction term = 0.95). The results were not meaningfully different in the univariate models (Table S2 in File S1).

## Discussion

In this study, we have identified, among genetic polymorphisms known to increase the risk of MS, several that also may play a role in the CNS location of early MS attacks. While the confidence intervals are wide, such that the associations should not be over-interpreted, the results are intriguing from a scientific standpoint. Both the *IL12A* and the *IRF8* polymorphisms were associated with spinal cord involvement. Secreted by dendritic cells and monocytes, IL12 stimulates IFNγ and suppresses T_H_2 cells [20]. The initiation of treatment with an approved MS disease-modifying therapy, interferon beta-1a, leads to increased expression of IL12 [21]. In light of these data, that the *IL12A* polymorphism is associated with increased spinal cord involvement is of interest given our previous work showing that patients treated with interferon beta were more likely to experience spinal cord involvement during attacks, an association that was independent of having had prior attacks involving the spinal cord [1], [22]. In contrast, subgroup analyses of the CHAMPS study showed that treatment with interferon seemed to confer the strongest protective effect against conversion to MS after the first attack in those whose first attack localized to the spinal cord [23]. The exact role that IL12 plays in the frequency and location of MS attacks, as well as the functional consequences of the polymorphism known to be associated with MS risk, thus demands further investigation.

The connection between polymorphisms in the other genes identified here and altered MS attack location is less clear. *IRF8* has been associated with regulating innate immunity, with B cell development, and with the differentiation of CD8+ T cells [24]. IL7 is important to many immune functions, including T cell differentiation and maintenance, and the *IL7R* polymorphism confers a functional change; specifically, it leads to an increase in the soluble (versus membrane-bound) form of the IL7R α chain [25]. Finally, the *CD6* gene product is involved in continuing the activation of T cells; CD4+ T cell proliferation is reduced in healthy people with two copies of the risk allele [26]. Alternatively, the polymorphisms may influence MS attack location based on local molecular properties in these regions, such as differential expression of adhesion blood-brain barrier molecules, similar to the finding that aquaporin-4 protein is located in the spinal cord, hypothalamus, and optic nerve, driving the involvement of these regions in neuromyelitis optica. The genes could also have roles that vary depending on CNS location.

Limitations of the study include that the results may not be generalizable to non-white patients with MS. That some ONTT patients were missing detailed data regarding second attack locations may have introduced some bias. Also, in some of the models, the 95% CIs included 1.0 even when a convincing direction of association emerged. Because genotyping for this study occurred prior to the publication of the 2011 MS genome-wide association study [27], we were unable to evaluate some of the susceptibility SNPs identified in that study for their association with relapse location. Finally, although the factors all have some *a priori* plausibility for influencing the phenotype of MS due to their known influence on susceptibility, it is possible that these findings are still due to chance given the number of analyses. Because this was a preliminary study in which the goal was to explore, rather than definitively ascertain, the associations between the SNPs and relapse location, we were more interested in evaluating the magnitudes and directions of the effects seen rather than only focusing on the p-values and performing adjustments for multiple comparisons. Our results thus need replication in a large, independent dataset, ideally including the more recently-identified MS risk alleles at the time such a study is conducted [27].

The location of early MS attacks within the CNS does not appear to be random. Here, we have identified that some of the genes important to MS susceptibility also influence which anatomic structures are affected. While the interplay between these genes and other as yet unknown factors important to attack location is not completely clear, the results presented here add to the growing support for utilizing genetics to predict disease outcomes in MS and to tailor therapies, based on those predictions, to each individual with the disease.

## Supporting Information

File S1 Table S1. Non-HLA genes associated with MS as of 2010. Table S2. Univariate predictors of location.
(DOCX)
