# Supplementary material for: Association of Multiple Sclerosis Susceptibility Variants and Early Attack Location in the CNS
Source: PLoS One. 2013 Oct 10;8(10):e75565. doi: 10.1371/journal.pone.0075565 (PMC3794979; doi:10.1371/journal.pone.0075565)
Supplement: File S1 — Table S1. Non-HLA genes associated with MS as of 2010. Table S2. Univariate predictors of location. (DOCX) [file pone.0075565.s001.docx]

Table S1. Non-HLA genes associated with MS as of 2010

| Gene  (Polymorphism) | Minor allele | Possession of at least one copy of minor allele, n (%) | Possession of at least one copy of risk allele, n (%) | MS risk allele |
| --- | --- | --- | --- | --- |
| *IL12A* (rs4680534) | C | 302 (60) | 302 (60) | C |
| *TMEM39A* (rs1132200) | A | 115 (23) | 496 (99) | G |
| *IL7R* (rs6897932) | T | 210 (42) | 476 (95) | C |
| *IL2RA* (rs2104286) | G | 184 (37) | 482 (96) | A |
| *CD6* (rs17824933) | G | 233 (46) | 233 (46) | G |
| *MPHOSPH9* (rs1790100) | G | 217 (43) | 217 (43) | G |
| *TNFRSF1A* (rs4149584) | T | 41 (8) | 41 (8) | T |
| *IRF8* (rs17445836) | A | 165 (33) | 484 (96) | G |
| *GPC5* (rs727986) | C | 203 (40) | 203 (40) | C |
| *CD58* (rs2300747) | G | 94 (19) | 494 (98) | A |
| *RGS1* (rs2760524) | A | 150 (30) | 492 (98) | G |
| *EVI5* (rs10735781) | C | 419 (83) | 330 (66) | G |
| *KIF21B* (rs12122721) | A | 250 (50) | 473 (94) | G |
| *CLEC16A* (rs12708716) | G | 286 (57) | 452 (90) | A |
| *CD226a* (rs763361) | C | 354 (70) | 363 (72) | T |
| *TYK2* (rs34536443) | C | 25 (5) | 478 (95)* | G |

*All subjects had at least one copy of the MS risk allele, so the number (%) of patients who had two copies (versus one) is presented here.

Table S2. Univariate predictors of location

|  | Spinal cord | Brainstem/cerebellum | Optic nerve |
| --- | --- | --- | --- |
| *IL12A* | 1.43 (1.06, 1.94), p=0.020 | 0.75 (0.54, 1.05), p=0.095 | 0.87 (0.62, 1.22), p=0.42 |
| *TMEM39A* | 2.34 (0.91, 6.04), p=0.077 | 3.15 (0.46, 21.35), p=0.24 | 0.18 (0.04, 0.89), p=0.035 |
| *IL7R* | 1.22 (0.57, 2.61), p=0.60 | 0.53 (0.24, 1.17), p=0.12 | 1.05 (0.51, 2.19), p=0.89 |
| *IL2RA* | 0.62 (0.33, 1.18), p=0.15 | 1.22 (0.58, 2.54), p=0.60 | 0.96 (0.43, 2.12), p=0.92 |
| *CD6* | 1.17 (0.86, 1.58), p=0.32 | 1.12 (0.80, 1.56), p=0.51 | 0.73 (0.52, 1.01), p=0.059 |
| *MPHOSPH9* | 0.95 (0.70, 1.29), p=0.74 | 1.20 (0.86, 1.67), p=0.28 | 0.82 (0.59, 1.15), p=0.25 |
| *TNFRSF1A* | 1.47 (0.89, 2.41), p=0.13 | 0.64 (0.36, 1.12), p=0.11 | 0.86 (0.49, 1.53), p=0.61 |
| *IRF8* | 2.01 (0.88, 4.59), p=0.098 | 0.79 (0.39, 1.58), p=0.50 | 0.85 (0.35, 2.02), p=0.71 |
| *GPC5* | 0.72 (0.53, 0.98), p=0.036 | 1.02 (0.73, 1.43), p=0.91 | 1.31 (0.94, 1.83), p=0.12 |
| *HLA-DRB1* | 0.93 (0.69, 1.26), p=0.65 | 1.03 (0.74, 1.44), p=0.85 | 1.13 (0.81, 1.57), p=0.47 |
| *CD58* | 0.59 (0.20, 1.75), p=0.34 | 0.77 (0.23, 2.56), p=0.68 | 1.27 (0.41, 3.90), p=0.67 |
| *RGS1* | 1.17 (0.45, 3.05), p=0.75 | 0.55 (0.19, 1.61), p=0.28 | 1.03 (0.36, 2.96), p=0.96 |
| *EVI5* | 0.97 (0.71, 1.34), p=0.87 | 1.11 (0.78, 1.60), p=0.56 | 0.83 (0.59, 1.17), p=0.29 |
| *KIF21B* | 1.46 (0.77, 2.78), p=0.25 | 0.94 (0.45, 1.96), p=0.87 | 0.90 (0.46, 1.76), p=0.76 |
| *CLEC16A* | 0.96 (0.57, 1.61), p=0.88 | 0.76 (0.45, 1.28), p=0.31 | 1.25 (0.72, 2.17), p=0.43 |
| *CD226a* | 1.08 (0.77, 1.51), p=0.66 | 0.92 (0.64, 1.33), p=0.66 | 1.09 (0.75, 1.56), p=0.66 |
| *TYK2* | 1.46 (0.64, 3.37), p=0.37 | 1.07 (0.44, 2.60), p=0.88 | 0.61 (0.28, 1.34), p=0.22 |

Results presented as odds ratios (95% confidence intervals), p values. The analyses take into account first and second attack locations. Of the entire cohort (n=503), 349 had a second attack. Attack location could not be resolved for 3 initial attacks and 47 second attacks.
